# Long-Term Outcomes Following Hepatic Resection and Radiofrequency Ablation of Colorectal Liver Metastases

**DOI:** 10.1155/2009/346863

**Published:** 2010-02-01

**Authors:** Andrew McKay, Katherine Fradette, Jeremy Lipschitz

**Affiliations:** ^1^Department of Surgery, Health Sciences Centre, University of Manitoba, Winnipeg, MB, Canada R3A 1R9; ^2^Department of Community Health Sciences, University of Manitoba, Winnipeg, MB, Canada R3A 1R9; ^3^Epidemiology and Cancer Registry, CancerCare Manitoba, Winnipeg, MB, Canada R3E 0V9

## Abstract

Recently some have called for randomized controlled trials comparing RFA to hepatic resection, particularly for patients with only a few small metastases. The objectives were to compare local recurrence and survival following RFA and hepatic resection for colorectal liver metastases. This was a retrospective review of open RFA and hepatic resection for colorectal liver metastases between January 1998 and May 2007. All patients who had RFA were considered to have unresectable disease. 58 patients had hepatic resection and 43 had RFA. A 5-year survival after resection was 43% compared to 23% after RFA. For patients with solitary lesions, a 5-year survival was 48% after resection and 15% after RFA. Sixty percent of patients suffered local recurrences after RFA compared to 7% after hepatic resection. RFA is inferior to resection. The results observed in this study support the consensus that RFA cannot be considered an equivalent procedure to hepatic resection.

## 1. Introduction

Hepatic resection is a major surgical intervention with significant potential complications risk. Much effort has been placed on developing regional ablative techniques such as radiofrequency ablation (RFA) [1, 2] in hopes of achieving similar survival as with hepatic resection, but with less morbidity. However, the outcomes following RFA have not been firmly established. While some authors have reported that local recurrence rates with RFA are not significantly different than those with anatomic or wedge resections of the liver [3], the current literature reports a wide range of local recurrence rates for colorectal liver metastases treated with RFA. These rates range from 5% [4] to as high as 39% [5, 6]. Reports of low recurrence rates may be a function of patient selection (i.e., small lesions) or short follow-up in some instances, and many series have combined patients with primary and secondary hepatic malignancies. To date, long-term survival remains difficult to interpret. In all these studies, patients were considered to have unresectable disease, which limits comparisons to patients undergoing hepatic resection.

Several studies have reported favorable survival rates and this has prompted some authors to call for randomized controlled trials comparing RFA to hepatic resection [7–9]. Other results have been much less optimistic [10]. An updated report from the same center [11] showed that with longer follow-up, the proportion of patients treated with RFA that had recurrence at the ablation site had risen from 9% to 37%. Furthermore, the 5-year overall survival for patients who had RFA was significantly inferior to those who had resection, even though patient characteristics and the proportion of patients receiving adjuvant chemotherapy were similar between groups.

The effectiveness of RFA remains controversial, and in the absence of randomized studies several questions remain. Patient selection is an obvious concern and publication bias may be present.

The hypothesis of this study is that treatment with RFA leads to significantly higher recurrence rates and decreased survival compared to surgical resection. If this is confirmed, then this will provide important evidence that will be helpful in guiding treatment decisions for patients with potentially resectable colorectal liver metastases.

The objectives of this study are to report the local recurrence rates and overall survival rates following open RFA and following hepatic resection for the treatment of colorectal liver metastases in the Province of Manitoba.

## 2. Materials and Methods

### 2.1. Study Design

This was a retrospective review of the outcomes of RFA and hepatic resection for colorectal liver metastases. The primary outcome measures were recurrence rates and overall survival. The study was approved by the Health Research Ethics Board at the University of Manitoba.

### 2.2. Subjects

The study was conducted from January 1998, the year when RFA first became available in Manitoba, until June 2007. All patients who underwent open RFA and/or surgical resection for colorectal liver metastases at either Health Sciences Centre (HSC) or St. Boniface General Hospital (SBGH), the two University-affiliated teaching hospitals in the Province, were included in the study. All major hepatic surgery in the Province was performed at these two hospitals; thus, it was a population-based study. The population of the Province is just over 1.1 million people and the catchment area of the two hospitals is slightly larger than that. Patients received systemic chemotherapy at CancerCare Manitoba (CCMB), an outpatient oncology centre for the province.

### 2.3. Procedure

All patients had previously underwent hepatic resection or RFA. All patients who had hepatic resection first had intraoperative ultrasound performed. In the early years of the study period, parenchymal transection was done with the “clamp-crush” method and with the Cavitron Ultrasonic Surgical Aspirator (CUSA; Integra Life Sciences).

All patients who underwent RFA were considered to have unresectable disease. Due to the retrospective nature of this analysis, it is not possible to determine the exact reason why patients were considered to have unresectable disease in each case. However, in general patients were considered to have unresectable disease because of extensive disease that would result in an insufficient liver remnant, proximity to critical structures, prohibitive comorbidity, or patient refusal. RFA was performed as an open procedure in all cases. An open approach was chosen over a percutaneous approach because of the added benefit of intraoperative ultrasound to discover unsuspected disease [12, 13], the potentially lower recurrence with an open technique [14], the ability to protect adjacent structures, and the ability to perform simultaneous resection in select cases. RFA was done under real-time ultrasound guidance using the RF 3000 Radiofrequency Ablation System (Boston Scientific) with either a 3 cm or 4 cm probe. The RFA tract was routinely ablated as the probe was withdrawn.

Systemic chemotherapy was administered at the discretion of the medical oncologists. Towards the end of the study period, chemotherapy was generally offered to all patients who were medically fit. Towards the end of the study period chemotherapy usually consisted of 5-fluourouracil and leucovorin in combination with either irinotecan or oxaliplatin, but a variety of regimens were used.

Eligible patients were later identified from the Medical Records Departments of both HSC and SBGH. The hospital charts and the outpatient charts at CCMB were then reviewed for demographic information, patient factors including comorbidities, pathological features of the primary tumors, dates of diagnoses, and the treatments received. The characteristics of the tumors including the number, distribution, and size of lesions were analyzed along with the nature and extent of the operative procedures. The outpatient charts were reviewed for dates and locations of disease recurrences. The Manitoba Health Population Registry is an administrative database belonging to Manitoba Health (the government agency that provides health insurance for all Manitobans). It lists up-to-date vital statistic, migration and loss to follow-up information for all people living in the Province of Manitoba, and was accessed in order to obtain the most accurate survival information possible.

Postoperative morbidity was graded according to a previously validated classification system [15]. Mortality was defined as either 30-day mortality or in-hospital mortality if patients died in hospital beyond 30 days. This is because mortality from postoperative liver failure in major hepatic surgery may occur well beyond 30 days [16], which is a commonly used end-point.

The outcomes for patients who underwent RFA, RFA plus hepatic resection, and hepatic resection were reported. The analysis focused mainly on comparing those who underwent hepatic resection to those who underwent RFA. Although, it was hypothesized that the limiting factor in terms of survival and recurrence in those who underwent simultaneous RFA and resection would be the effectiveness of the RFA, this group was excluded from the analyses to prevent any confounding effect. A subgroup analysis comparing outcomes of patients with solitary metastases who underwent RFA to those who underwent hepatic resection was planned a priori.

### 2.4. Sample Size

It was planned to include all eligible patients treated during the study period. A sample size calculation was performed to verify that the sample would be adequately powered for our primary objective of assessing local recurrence. It was anticipated that the local recurrence rate of lesions treated by RFA would be approximately 40%, while the recurrence rate for lesions treated by resection would be under 10% [11]. With a *P*-value of  .05, 32 patients in each group were needed to detect this difference with a power of .80. The number of eligible patients in each group considerably exceeded this number, so the study was more than adequately powered to detect this endpoint.

### 2.5. Statistical Analysis

Overall survival and disease-free survival were calculated from the date of surgical intervention. Continuous variables were analyzed with Student's *t* test, and categorical variables were analyzed with a chi-square or Fisher's Exact Test where appropriate. Survival and recurrence rates were calculated using the Kaplan-Meier method and comparisons between groups were done with the logrank test. Predictors of overall and disease-free survival were analyzed by performing a Cox Proportional Hazards regression model using a backwards selection process. A *P*-value of .05 was used to define statistical significance.

## 3. Results

### 3.1. Patients

During the study period, 58 patients underwent hepatic resection, 43 underwent RFA, and 12 underwent simultaneous hepatic resection and RFA for colorectal liver metastases. Table 1 shows the patient characteristics in each group. Three patients were lost to follow-up, either because they lived out of province or moved out of province during the study period. The mean and median follow-up duration for all patients was 38 months and 33 months, respectively. The median follow-up duration for those who had resection, RFA, and both resection and RFA was 25 months (range 4 to 106), 42 months (range 15 to 85), and 20 months (range 15 to 45), respectively. There were no significant differences between groups.

### 3.2. Procedure and Complications

Sixty-eight percent of patients in the group who had liver resection alone had major resections (a lobectomy or greater). The operative time was significantly longer for the resection group compared to the RFA group (median of 269 minutes (range 118 to 452) versus 204 minutes (range 113 to 316); *P* < .0005). Operative blood loss (median 1400 mL (range 100 to 9000) versus 150 mL (range 50 to 2300); *P* < .0005) and transfusion requirements (44% of patients versus 5%; *P* < .0005) were higher for resection than for RFA. Rates of ICU admission (6.5% of patients overall), and length of stay (median of 7 days for all patients; range 1 to 48) were not significantly different between patients having hepatic resection and RFA. More patients developed complications with resection compared to RFA (59% compared to 43%), but the difference was not statistically significant. Overall, 32% of all complications were major (Grade III or higher), and there was no difference between groups. There was only one postoperative death, which occurred in a patient who underwent RFA alone.

### 3.3. Overall Survival

**Figure 1:**
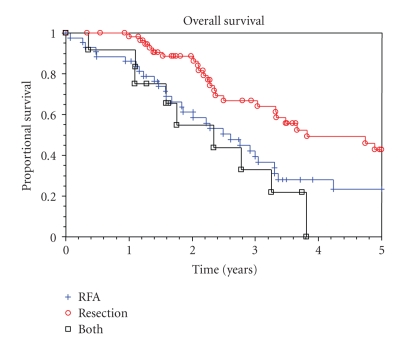
Overall survival.

The median survival for patients who had resection, RFA, and resection in combination with RFA of their CRC metastases was 3.8 years (95% CI = 3.0 to 5.9 years), 2.6 years (95% CI = 1.8 to 3.3 years), and 2.3 (95% CI = 1.6 to 3.2 years), respectively. The 5-year overall survival after resection alone was 43% (95% CI = 26 to 58%), while the 5-year survival after RFA alone was 23% (95% CI = 11 to 39%), as shown in Figure 1. This difference was statistically significant (*P* = .02). When comparing the overall survival in patients who underwent surgical resection to those who had RFA alone, the following variables were significant on univariate analysis (see Table 2): which procedure was performed, age (less than 70 versus 70 or greater), size of metastasis (under 5 cm versus 5 cm or greater), number of lesions (less than 5 versus 5 or more), and the timing of the lesion (patients with synchronous lesions had better survival compared to patients with metachronous disease). In the multivariate analysis, age was no longer a significant predictor of survival. The other variables remained significant (Table 3). The majority of patients (67%) received postoperative chemotherapy, but this was not significantly associated with increased survival.

Thirty-two patients underwent repeat surgical procedures for recurrent disease in their liver and/or lungs. Seven patients underwent a subsequent hepatic resection and 13 patients underwent a subsequent RFA procedure. Three of the patients who underwent an additional RFA for recurrence also underwent a pulmonary resection for metastases. Another 12 patients underwent pulmonary resections without repeat liver procedures.

**Figure 2:**
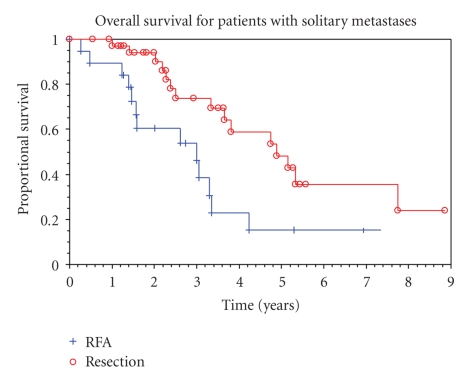
Overall survival for patients with solitary metastases.

A preplanned subgroup analysis was performed for patients with solitary lesions treated by resection versus RFA. The median overall survival times in this subset for resection and for RFA were 4.9 years (95% CI = 3.7 to 7.7 years) and 3.0 years (95%  CI 1.6 to 3.4 years), respectively. The 5-year survival rates were 48% (95% CI = 26 to 67%) and 15% (95% CI = 2.6 to 38%), respectively. The overall survival for patients with solitary liver lesions treated by resection compared to RFA is shown in Figure 2. The size of the solitary lesion (under 5 cm versus 5 cm or greater) and the procedure performed were independent predictors of overall survival (Table 4). Even when limited to solitary lesions less than 3 cm in diameter, the survival associated with resection was significantly greater than with RFA.

### 3.4. Disease-Free Survival and Recurrence

The median times to recurrence for patients who had hepatic resection, RFA, and resection plus RFA were 11 months (range 2 to 49), 7 months (range 1 to 26), and 8 months (range 2 to 30), respectively. The 5-year disease-free survival (DFS) for patients who underwent hepatic resection was 17% (95% CI = 7 to 29%), compared to 15% (95% CI = 6 to 28%) for patients who underwent RFA alone (*P* = .06). In a multivariate regression the procedure performed, the size of lesion, the number of lesions, and the hospital where the surgery was performed were independent predictors of disease-free survival. 

**Figure 3:**
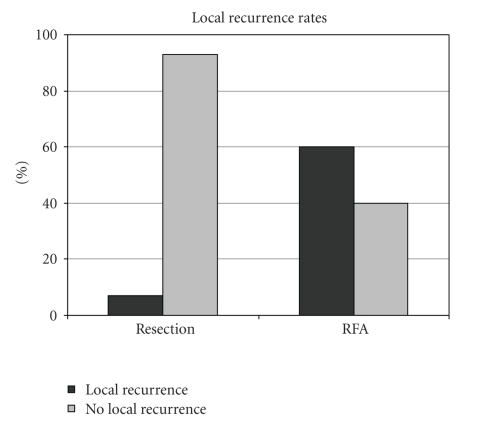
Local recurrence rates.

The rates of local recurrence were dramatically different (Figure 3). Over the course of the study (mean follow-up of 46 months for patients undergoing RFA), 60% of patients who had only open RFA suffered local recurrences compared to 7% of patients who underwent hepatic resection (*P* < .0005). Over the study period, the local recurrence rate dropped, and in the last 4 years of the study period it was 43%. The local recurrence rate in 10 patients with small (3 cm or smaller), solitary lesions treated by RFA was still 50%.

## 4. Discussion

This is a population-based study reporting the experience with RFA and hepatic resection in the Province of Manitoba, Canada. By accessing provincial vital statistics information from the Government, the survival figures in this study are thought to be quite accurate. The 5-year survival was 43% (95% CI = 26% to 58%) and the median survival was 3.8 years (95% CI = 3.0 to 5.9 years) following hepatic resection in this study. This generally compares favorably to other reports in the literature [17–19], suggesting that these results are generalizable. The number of metastases and the size of metastases were found to be independent predictors of survival, as seen in other reports [17]. Chemotherapy use was not standardized in the study, but was not found to be a predictor of increased survival on univariate or multivariate analysis.

The unexpectedly high local recurrence rate with RFA is an alarming finding. With a median follow-up of 42 months, patients who underwent RFA alone had a local recurrence rate of 60%. This is higher than what is reported elsewhere in the literature and may be due to several causes. Firstly, the median length of follow-up in this study is longer than most other reports. Other studies report recurrence rates from 6% to 39% with follow-up ranging from 6 to 28 months [3, 4, 6, 7, 10, 20–28]. Abdalla et al. reported a local recurrence rate of 9% after RFA for colorectal liver metastases with a follow-up of 21 months [10]. When they updated their experience with a subgroup of patients with solitary lesions receiving RFA, the local recurrence rate rose to 37% when the follow-up had lengthened to a median of 31 months [11]. Perhaps with longer follow-up, the local recurrence rate in their series may have been even higher.

Another reason for this high local recurrence rate is that this study represents the initial experience with this technology in the Province of Manitoba. Part of the high rate may be due to a learning curve, which seems to be present over the first 40 to 50 cases [14, 29, 30]. Furthermore, the selection criteria over the time frame of this study changed. In the early years of the study, lesions as large as 6 to 7.5 cm in diameter were being ablated. Treatment of such large lesions is associated with very high recurrence [5, 14, 31] and most centers would restrict RFA to smaller lesions. In fact, there was a trend to ablation of smaller lesions over the course of the study, but the trend was not statistically significant (*P* =  .077). Similarly, there was a nonsignificant trend to treat lower number of lesions per single patient over time in the present study, since a high number of lesions are a risk factor for local recurrence [2]. Both the number and size of metastases were significant predictors of overall survival and disease-free survival in the multivariate analyses in this study. Patients with lesions over 5 cm in size and/or with more than 4 or 5 lesions would generally no longer be treated by RFA in Manitoba. In addition, many patients were considered to have unresectable disease due to proximity to critical vascular structures, which may have acted as a “heat-sink.” A more aggressive surgical approach has been adopted in the recent years and it is likely that many such patients would now undergo resection. It is likely that with more experience and improvements in selection criteria, the recurrence rates will fall in the future. Towards the end of the study period, the local recurrence rate did drop to 43%, which is much closer to what has been reported from other larger centers [11, 32]. This is likely due to more prudent patient selection, although the shorter follow-up for these later patients may also play a role.

There are other reports of very high local recurrence following RFA of colorectal liver metastases in the recent literature as well. In one of the largest series, Berber and Siperstein [32] recently reported a local recurrence rate for colorectal lesions of 34% with a median of 12 months of follow-up. Other reports suggest the recurrence rate may be close to 40% [5, 6, 11]. Therefore, while a local recurrence rate of 60% seems inappropriately high, the changes in patient selection and techniques that have evolved are not expected to reduce recurrence to the range seen following hepatic resection.

RFA was associated with worse survival and recurrence compared to hepatic resection in all analyses in this study. Others have also shown inferior survival with RFA compared to resection. In a series of 418 patients with colorectal metastases, Abdalla et al. [10] reported a 4-year survival following hepatic resection of 65%. This was significantly higher than the 36% 4-year survival following resection plus RFA and the 22% 4-year survival following RFA alone. 

Some studies have shown more promising results. In a series of 45 consecutive patients with solitary colorectal liver metastases Oshowo et al. [8] reported almost identical 3-year survival following RFA compared to resection (53% and 55%, resp.). A recent report from Berber and Siperstein [33] described their experience with 158 patients who underwent laparoscopic RFA and 90 patients who underwent open resection of solitary colorectal liver metastases. The actual 5-year survival was 30% for RFA and 40% for resection, which was not statistically different. However, the 3-year survival for RFA in patients without extrahepatic disease was 35% compared to 70% for those who had liver resection. 

All patients in the current series and in the other series listed above who underwent RFA were considered to have unresectable disease, and consequently there must be some selection bias present. It is very likely that there were inherent differences in the biology and aggressiveness of the tumors' behavior between the two groups. Because the study is retrospective, it is impossible to completely control for these differences. In this study patients who underwent RFA more often had multiple lesions and bilateral lesions. In addition, RFA is offered more commonly to patients with multiple medical comorbidities, which may also bias survival in favor of resection. These fundamental differences will always be a limitation in interpreting the results of such studies that show inferior results with RFA in patients with unresectable disease compared to hepatic resection in patients with resectable disease. A randomized trial with very strict inclusion criteria would be needed to eliminate this weakness [7–9]. However, the high local recurrence rates following RFA observed in this study and others [10, 11] are difficult to ignore. Even with solitary lesions less than 3 cm in diameter, the recurrence rate with hepatic resection appears to be superior to that of RFA.

## 5. Conclusions

With the 0% operative mortality rate for hepatic resection in this study and the very low mortality reported by others in the literature [17, 18, 34], the improved safety of liver surgery is well established. Until current RFA technology improves or alternative ablation technology is developed with much improved local recurrence rates, it would be very difficult to support a randomized trial. The results observed in this study support the consensus that RFA cannot be considered an equivalent procedure to hepatic resection. Resection must be considered the standard of care for colorectal metastases.

## Figures and Tables

**Table 1:** Overall patient characteristics.

		All patients	Resection	RFA	Both	*P*-value (Res versus RFA)*

*n*		113	58	43	12	
Age	Median	67	67	67	63	NS
	Range	28 to 83	28 to 83	37 to 83	45 to 82	
Gender	M	61	29	25	7	NS
	F	52	29	18	5	
Primary site	Colon	80	44	29	7	NS
	Rectal	33	14	14	5	
ASA score	Median	2	2	2	2	NS
	Range	1 to 3	1 to 3	2 to 3	2 to 3	
Timing of primary	Synchronous	51	24	21	6	NS
	Metachronous	62	34	22	6	
Node Pos primary^†^	Yes	69	33	27	9	NS
	No	34	20	13	1	
No. lesions	Median	1	1	2	3.5	NS
	Range	1 to 7	1 to 7	1 to 6	1 to 5	
Solitary lesion	Yes	58	37	19	0	<.001
	No	54	20	24	12	
Size (cm)	Median	4	4.1	3	4.8	.012
	Range	1 to 14.5	1.5 to 14.5	1 to 7.5	1.2 to 7	
Bilateral disease^†^	Yes	22	2	13	7	<.001
	No	31	17	11	5	
Preop CEA (mg/L)^†^	Median	18.1	24	18.1	6.1	NS
	Range	0 to 699	0 to 279	1 to 699	2 to 58	

*P-value for comparison between patients undergoing resection alone compared to those undergoing RFA alone.

^†^There are missing values for some patients for the marked variables.

**Table 2:** Results of univariate analysis of predictors of overall survival.

		Med OS (Yr)	5-yr OS (%)	Hazard ratio	95% confidence interval	*P*-value	
Procedure	RFA	2.6	23	1.00			
	Resection	3.8	43	0.54	0.32	.91	.021
Age	<70	3.5	38	1.00			
	≥70	2.5	26	1.81	1.08	3.05	.025
Gender	Male	3.1	30	1.00			
	Female	3.4	38	0.87	0.51	1.51	.630
Hospital	HSC	3.3	30	1.00			
	SBGH	3.3	40	1.04	0.60	1.78	.900
Primary tumor	Colon	3.3	36	1.00			
	Rectum	2.5	28	1.13	0.63	2.00	.660
Node positive primary	No	3.1	44	1.00			
	Yes	3.3	30	0.97	0.54	1.72	.910
Timing of lesion(s)	Synchronous	3.8	50	1.00			
	Metachronous	2.4	19	1.99	1.17	3.39	.012
Bilateral disease	Yes	2.4	36	1.00			
	No	3.3	32	0.78	0.40	1.57	.460
No. lesions	<5	3.3	37	1.00			
	≥5	2.1	0	2.93	1.30	6.60	.009
Size of lesion(s)	<5 cm	3.5	44	1.00			
	≥5 cm	2.5	11	1.85	1.09	3.17	.024
Postoperative chemo	Yes	3.0	24	1.00			
	No	3.3	39	0.85	0.49	1.45	.540

**Table 3:** Results of multivariate Cox Proportional Hazards regression model of predictors of overall survival.

		Hazard ratio	95% confidence		*P*-value
			interval		
Procedure	RFA	1.00			
	Resection	0.36	0.19	0.70	.002
Size of lesion(s)	<5 cm	1.00			
	≥5 cm	2.43	1.26	4.67	.008
No. lesions	<5	1.00			
	≥5	6.08	2.21	16.70	<.001
Timing of lesion(s)	Synchronous	1.00			
	Metachronous	2.92	1.50	5.70	.002

**Table 4:** Results of multivariate Cox Proportional Hazards regression model of predictors of overall survival in patient with solitary liver metastases.

		Hazard ratio	95% confidence		*P*-value
			interval		
Procedure	RFA	1.00			
	Resection	0.38	0.18	0.81	.013
Size of lesion(s)	<5 cm	1.00			
	≥5 cm	3.06	1.43	6.55	.004
Timing of Lesion(s)	Synchronous	1.00			
	Metachronous	3.36	1.39	8.14	.007

## Abbreviations

RFA: Radiofrequency Ablation
HSC: Health Sciences Centre
SBGH: St. Boniface General Hospital
CCMB: CancerCare Manitoba.
